# Supplementary material for: MetaRibo-Seq measures translation in microbiomes
Source: Nat Commun. 2020 Jun 29;11:3268. doi: 10.1038/s41467-020-17081-z (PMC7324362; doi:10.1038/s41467-020-17081-z)
Supplement: Supplementary file 10 — Supplementary Data 7 [file 41467_2020_17081_MOESM10_ESM.zip › File2/Confidence_High_Taxonomy/79804_out.krona.html]

Javascript must be enabled to view this page.

members
magnitude
magnitudeUnassigned
count
unassigned
taxon
rank

79804\_out

13

13
2
superkingdom

phylum
13
976

13
200643
class

171549
13
1
order

SRS021484\_contig\_number\_contig-100\_2283.171187

family
171550
2

239759
2
genus


SRS144135\_contig\_number\_contig-100\_299.205043SRS146812\_contig\_number\_6188
1118061
2
species

1853231
1
family

574697
1
genus

2042309

SRS143070\_contig\_number\_4651
1
species

8
815
family


SRS014683\_contig\_number\_contig-100\_1881.112963SRS016018\_contig\_number\_16818SRS049995\_contig\_number\_9739SRS077641\_contig\_number\_contig-100\_577.130653
genus
4
816
8

species
1

SRS052576\_contig\_number\_contig-100\_847.16356
310297

species
28116

SRS015190\_contig\_number\_contig-100\_293.101226SRS024663\_contig\_number\_2189
2

species

SRS050752\_contig\_number\_25822
469590
1

family
2005525
1

genus
1
375288

species

SRS892882\_contig\_number\_12463
658662
1
